# Oral amoxicillin plus gentamicin regimens may be superior to the procaine-penicillin plus gentamicin regimens for treatment of young infants with possible serious bacterial infection when referral is not feasible: Pooled analysis from three trials in Africa and Asia

**DOI:** 10.7189/jogh.12.04084

**Published:** 2022-11-21

**Authors:** Adrien Lokangaka Longombe, Adejumoke Idowu Ayede, Irene Marete, Fatima Mir, Clara Ladi Ejembi, Mohammod Shahidullah, Ebunoluwa A Adejuyigbe, Robinson D Wammanda, Antoinette Tshefu, Fabian Esamai, Anita K Zaidi, Abdullah H Baqui, Simon Cousens

**Affiliations:** 1 Department of Community Health, Kinshasa School of Public Health, Kinshasa, DR Congo; 2 College of Medicine, University of Ibadan, and University College Hospital, Ibadan, Nigeria; 3 Department of Child Health and Paediatrics, School of Medicine, Moi University, Eldoret, Kenya; 4 Department of Pediatrics and Child Health, The Aga Khan University, Karachi, Pakistan; 5 Department of Community Medicine, Ahmadu Bello University Teaching Hospital, Ahmadu Bello University, Zaria, Nigeria; 6 Bangabandhu Sheikh Mujib Medical University, Dhaka, Bangladesh; 7 Department of Paediatrics and Child Health, Obafemi Awolowo University, Ile-Ife, Nigeria; 8 Department of Paediatrics, Ahmadu Bello University Teaching Hospital, Ahmadu Bello University, Zaria, Nigeria; 9 Bill and Melinda Gates Foundation, Seattle, Washington, United States of America; 10 Department of International Health, Johns Hopkins Bloomberg School of Public Health, Baltimore, Maryland, United States of America; 11 Department of Infectious Disease Epidemiology, London School of Hygiene and Tropical Medicine (LSHTM), London, United Kingdom

## Abstract

**Background:**

Hospital referral and admission in many- low and middle-income countries are not feasible for many young infants with sepsis/possible serious bacterial infection (PSBI). The effectiveness of simplified antibiotic regimens when referral to a hospital was not feasible has been shown before. We analysed the pooled data from the previous trials to compare the risk of poor clinical outcome for young infants with PSBI with the two regimens containing injectable procaine penicillin and gentamicin with the oral amoxicillin plus gentamicin regimen currently recommended by the World Health Organization (WHO) when referral is not feasible.

**Methods:**

Infant records from three individually randomised trials conducted in Africa and Asia were collated in a standard format. All trials enrolled young infants aged 0-59 days with any sign of PSBI (fever, hypothermia, stopped feeding well, movement only when stimulated, or severe chest indrawing). Eligible young infants whose caretakers refused hospital admission and consented were enrolled and randomised to a trial reference arm (arm A: procaine benzylpenicillin and gentamicin) or two experimental arms (arm B: oral amoxicillin and gentamicin or arm C: procaine benzylpenicillin and gentamicin initially, followed by oral amoxicillin). We compared the rate of poor clinical outcomes by day 15 (deaths till day 15, treatment failure by day 8, and relapse between day 9 and 15) in reference arm A with experimental arms and present risk differences with 95% confidence interval (CI), adjusted for trial.

**Results:**

A total of 7617 young infants, randomised to arm A, arm B, or arm C in the three trials, were included in this analysis. Most were 7-59 days old (71%) and predominately males (56%). Slightly over one-fifth of young infants had more than one sign of PSBI at the time of enrolment. Severe chest indrawing (45%), fever (43%), and feeding problems (25%) were the most common signs. Overall, those who received arm B had a lower risk of poor clinical outcome compared to arm A for both per-protocol (risk difference = -2.1%, 95% CI = -3.8%, -0.4%; *P* = 0.016) and intention-to-treat (risk difference = -1.8%, 95% CI = -3.5%, -0.2%; *P* = 0.031) analyses. Those who received arm C did not have an increased risk of poor clinical outcome compared to arm A for both per-protocol (risk difference = -1.1%, 95% CI = -2.8%, 0.6%) and intention-to-treat (risk difference = -0.8%, 95% CI = -2.5%, 0.9%) analyses. Overall, those who received arm B had a lower risk of poor clinical outcome compared to the combined arms A and C for both per-protocol (risk difference = -1.6%, 95% CI = -3.5%, -0.1%; *P* = 0.035) and intention-to-treat (risk difference = -1.4%, 95% CI = -2.8%, -0.1%; *P* = 0.049) analyses.

**Conclusions:**

Analysis of pooled individual patient-level data from three large trials in Africa and Asia showed that the WHO-recommended simplified antibiotic regimen B (oral amoxicillin and injection gentamicin) was superior to regimen A (injection procaine penicillin and injection gentamicin) and combined arms A and C (injection procaine penicillin and injection gentamicin, followed by oral amoxicillin) in terms of poor clinical outcome for the outpatient treatment of young infants with PSBI when inpatient treatment was not feasible.

**Registration:**

AFRINEST study [9] is registered with the Australian New Zealand Clinical Trials Registry: ACTRN12610000286044. SATT Bangladesh study [10] is registered with ClinicalTrials.gov: NCT00844337. SATT Pakistan study [11] is registered at ClinicalTrials.gov: NCT01027429.

Of the estimated 2.5 million newborns who die each year, neonatal infections, including pneumonia, sepsis, and meningitis, are estimated to be the cause of 500 000 deaths [1]. Most of these deaths occur in Sub-Saharan Africa and South-East Asia [2]. Hospital-based injectable antibiotic treatment for at least 7 days is initiated based on clinical signs [3]. Unfortunately, hospital referral and admission are not feasible for many sick neonates/young infants in low-resource settings [4-7], resulting in deaths due to the absence of timely, appropriate treatment [8].

Considering this, a large, multi-stakeholder collaborative study was established to generate evidence to optimally treat young infants 0-59 days old who present with possible serious bacterial infection (PSBI) when treatment at a hospital is not feasible. These trials, termed the Simplified Antibiotics Treatment Trials (SATT), were conducted in Asia (Bangladesh and Pakistan) and in Africa (Democratic Republic of Congo, Kenya, and Nigeria – African Neonatal Sepsis Trial (AFRINEST)) [9-11]. Three antibiotic regimens were evaluated in all three trials, although AFRINEST also evaluated a fourth regimen (**Box 1**) [9-11]. All trials individually reported equivalence of treatment failure with the assessed regimens based on pre-determined criteria [9-11]. An additional trial for young infants 0-59 days old with only fast breathing was also carried out in the three African countries [12].

Box 1 **Treatment regimens in SATT and AFRINEST [9-11]**

The following treatment regimens were evaluated in the three trials:

1. Arm A- intramuscular daily injections of procaine penicillin and gentamicin for 7 days.

2. Arm B- oral amoxicillin and intramuscular gentamicin for 7 days.

3. Arm C- intramuscular procaine penicillin and gentamicin for 2 days followed by oral amoxicillin for another 5 days.

4. Arm D (Only in AFRINEST)- oral amoxicillin for 7 days plus intramuscular gentamicin only for the initial 2 days.

Antibiotic dosages:

1. Injection procaine benzylpenicillin in a dose of 50000 units/kg once per day intramuscularly.

2. Injection gentamicin in the range 4.0 mg/kg once per day IM in the first week of life and 7.5 mg/kg once daily intramuscularly thereafter.

Oral amoxicillin in suspension in a dose of 100 mg/kg per day (less than 2 kg are given 75 mg/kg per day), divided into two equal doses.

The World Health Organization (WHO) recommended simplified antibiotic regimens for young infants with PSBI signs based on the three trials [9-11] if parents refused to accept hospital referrals despite best efforts [13,14]. Some experts have questioned the statistical power of these trials, arguing that the PSBI cases were relatively mild and may be caused by a bacterial infection; therefore, true equivalence may not be harder to demonstrate. However, the results of the trials consistently showed lower rates of treatment failure in the currently WHO-recommended treatment arms compared to the other arms. In the per-protocol analysis (the primary analysis in the publications because of equivalence design), treatment failure rates with intramuscular gentamicin plus oral amoxicillin arms (arm B, recommended by WHO) were 6%, 8%, and 10%, compared with 8%, 10%, and 12% in the procaine penicillin plus gentamicin arm (arm A, not recommended by WHO) in AFRINEST, Bangladesh and Pakistan, respectively [9-11].

We conducted a pooled analysis across the three trials [9-11] to compare the risk of poor clinical outcome by day 15 of enrolment with currently WHO-recommended treatment regimens (arm B) compared with other regimens (arm A) and with combined arms A and C for PSBI when referral is not feasible. We aimed to obtain more precise estimates of the risk difference by comparing the currently WHO-recommended outpatient treatment regimens (arm B) with the trial reference regimen (arm A) and with combined arms A and C. We conducted per-protocol and intention-to-treat analyses.

## METHODS

Infant records from three individually randomised trials conducted in Africa and Asia were collated in a standard format. The details of the design of the methods used in each trial have been described in detail elsewhere [9-11,15-18]. In brief, one trial was performed across five sites in three countries (the Democratic Republic of Congo, Kenya, and Nigeria) in Africa (AFRINEST). The other two trials were conducted in Asia (Bangladesh and Pakistan).

### Design of trials and trial methods

All three trials enrolled young infants aged 0 to 59 days with at least one of the following five signs: fever (body temperature ≥38°C), hypothermia (body temperature <35.5°C), cessation of feeding well, movement only when stimulated, or severe chest indrawing. Young infants with signs of very severe disease, including convulsions, apnoea, inability to feed at all, unconsciousness, inability to cry, cyanosis, persistent vomiting or bulging fontanelle were excluded. Very low weight (<1500 g at the time of presentation) and hospital admission for illness in the past two weeks or previously enrolled in the study were also excluded. Caretakers of young infants clinically eligible for enrolment were advised a referral to a hospital for admission, and only young infants whose caretakers refused hospital admission and consented to inclusion in the trial were enrolled.

Following recruitment, young infants were randomised to one of the following three treatment arms in Bangladesh and Pakistan: arm A, the trial reference arm, arm B, and arm C, while AFRINEST also included a third alternative treatment arm D (**Box 1**). The trials were each designed to demonstrate that the “experimental” treatments (arms B, C, or D) were as effective as the reference treatment (arm A).

In all sites, young infants were assessed daily for the first seven days after enrolment (day 2-8) and on two other occasions during the second week after enrolment (day 11 and day 15). The primary outcome in all trials was treatment failure during the first week after enrolment, as defined in **Box 2**.

Box 2 **Definition of poor clinical outcomes**

1. Death from the day of enrolment till day 15

2. Treatment failure is the occurrence of any of the following:

- Clinical deterioration at any time up to Day 8 based on the presence of at least one of the following danger signs: unconscious, convulsions, unable to feed, apnoea, cyanosis, bulging fontanelle, major bleeding, persistent vomiting.

- Decision at any time up to day 8 by study personnel to change the antibiotic regimen or add another antibiotic for either of the following reasons:

- New-onset infectious co-morbidity (ie, severe omphalitis, bone or joint infection, or severe skin or soft tissue infection), or

- Serious non-fatal antibiotic-associated adverse event (ie, severe diarrhoea associated with dehydration; Stevens - Johnson syndrome; anaphylaxis; or, acute renal failure).

- Hospitalisation at any time up to Day 8 for any reason.

- On or after Day 3: the occurrence of new signs of PSBI (any of the following five signs: fever, hypothermia, poor feeding/poor suck, severe chest indrawing, movement only when stimulated). (A “new” sign was one that was not present at the time of enrolment.)

- On Day 4, for infants with multiple signs at enrolment: Presence of at least two of the signs that were present on enrolment;

- On Day 4, for infants with a single sign on enrolment: Presence of the same sign that was present on enrolment.

- On or after Day 5: Recurrence of any of the following signs: fever, hypothermia, severe chest indrawing, movement only when stimulated, or poor feeding. (Recurrence implies the presence of the sign on enrolment AND documented resolution of the sign on at least one follow-up visit with the subsequent reappearance of the same sign on at least one follow-up visit on or after Day 5.)

- Persistence on Day 8 of any of the five signs of PSBI that was present on enrolment.

3. Non-fatal relapse (after the disappearance of all signs of clinical severe infection by day 8, emergence of any sign of critical illness, or severe infection between days 9 and 15 after enrolment)

Young infants were defined as adherent to treatment provided that they received 100% of scheduled antibiotic doses on days 1-3 or by the time of treatment failure, they received at least 50% of scheduled doses of each antibiotic on days 4 to 7 or by the time of treatment failure, and they did not receive any non-study injectable antibiotic prior to the day 8 assessment or treatment failure and did not receive any non-study oral antibiotic on days 1-3 prior to any treatment failure.

In Bangladesh and Pakistan, young infants were classified as having adequate clinical follow-up if follow-up was completed during days 2-4, if follow-up was completed on at least one of days 5-8, and if the infant’s vital status on day 8 was known.

In AFRINEST, an infant was considered to have adequate clinical follow-up providing s/he was seen by an independent outcome assessor on day 4 and at least one of days 8, 11 and 15. In all trials, an infant was classified as eligible for inclusion in the per-protocol analysis providing that s/he was adherent to treatment and had an adequate clinical follow-up.

### Statistical analysis

The individual patient-level data, including the baseline characteristics, treatment regimen, and clinical outcome of each child, were pooled for the analyses. We excluded infants enrolled in arm D of the AFRINEST because arm D regimens were not considered in the Bangladesh and Pakistan trials. The primary aim was to obtain more precise estimates of the risk difference by comparing experimental treatment regimens (arm B or C) with standard regimens (arm A).

We performed both per-protocol and intention-to-treat analyses. We calculated the pooled risk difference with 95% confidence intervals (95% CI) and risk ratio with 95% CI for poor clinical outcomes between the experimental regimens (arm B or C) and arm A for outpatient treatment of young infants with PSBI, adjusting for trial. Additionally, we calculated the trial-specific risk difference with 95% CI and risk ratio with 95% CI for poor clinical outcomes between the experimental regimens (arm B or C) and arm A for outpatient treatment of young infants with PSBI. Moreover, we also calculated the pooled risk difference with 95% CI and risk ratio with 95% CI for poor clinical outcomes between arm B and combined arms A and C. All analyses were performed using Stata version 16 (www.stata.com).

## RESULTS

In three trials, 7617 young infants up to 2 months of age were enrolled, 2674 in Africa (excluding arm D) [9], 2490 in Bangladesh [10] and 2453 in Pakistan [11]. There were some variations in the characteristics of infants enrolled across the three trials (**Table 1**). Overall, nearly a third were younger than seven days at the time of enrolment, while this proportion was 44% in Pakistan and 10% in Bangladesh. The ratio of male and female infants was almost equal except in Bangladesh (males = 61.5%). The Bangladesh trial, which was primarily conducted in outpatient facilities of hospitals, had a higher proportion of infants with two or more inclusion signs (38.0%) than the community-based trials (Africa = 12.3%, Pakistan = 12.7%). The most common sign among enrolled infants was severe chest indrawing (45.5%), followed by fever/high body temperature (43.1%), cessation of feeding well (25.3%), hypothermia/low body temperature (5.6%), and movement only when stimulated (3.4%). Much higher proportions of underweight infants (weight-for-age Z-score<-2) were seen in Bangladesh (31.8%) and Pakistan (38.3%) than in Africa (16.9%), reflecting a higher prevalence of low birth weight infants in Asia.

**Table 1:** Selected characteristics of participants by trial*

Characteristics	AFRINEST	Bangladesh trial	Pakistan trial	Total
**Enrolled (N)**	2674	2490	2453	7617
**Infants <7 days**	872 (32.6%)	253 (10.2%)	1083 (44.2%)	2208 (29.0%)
**Male**	1403 (52.5%)	1530 (61.5%)	1309 (53.4%)	4242 (55.7%)
**Inclusion signs**				
One sign only	2345 (87.7%)	1543 (62.0%)	2141 (87.3%)	6029 (79.1%)
More than one sign	329 (12.3%)	947 (38.0%)	312 (12.7%)	1588 (20.9%)
Fever present	1231 (46.0%)	1035 (41.6%)	1015 (41.4%)	3281 (43.1%)
Hypothermia present	142 (5.3%)	52 (2.1%)	233 (9.5%)	427 (5.6%)
Severe chest indrawing present	1169 (43.7%)	1478 (59.4%)	818 (33.4%)	3465 (45.5%)
Feeding problem present	404 (15.1%)	920 (37.0%)	606 (24.7%)	1930 (25.3%)
Movement only when stimulated present	76 (2.8%)	57 (2.3%)	122 (5.0%)	255 (3.4%)
**Weight at enrollment <2500 g**	269 (10.1%)	256 (10.3%)	627 (25.6%)	1152 (15.1%)
**Weight for age Z-score<-2**	452 (16.9%)	793 (31.8%)	940 (38.3%)	2185 (28.7%)
**Facility delivery**	1257 (47.0%)	1070 (43.0%)	1223 (49.9%)	3550 (46.6%)
**Treatment arm**				
Arm A†	894 (33.4%)	830 (33.3%)	820 (33.4%)	2544 (33.4%)
Arm B‡	884 (33.1%)	831 (33.4%)	816 (33.3%)	2531 (33.2%)
Arm C§	896 (33.5%)	829 (33.3%)	817 (33.3%)	2542 (33.4%)
**Poor clinical outcome¶**				
Per-protocol	200 (7.9%)	250 (10.6%)	303 (13.5%)	753 (10.6%)
Intention-to-treat	208 (7.8%)	280 (11.2%)	333 (13.6%)	821 (10.8%)

AFRINEST – African Neonatal Sepsis Trial

*Data presented as n (%) unless otherwise specified.

†Arm A – intramuscular daily injections of procaine penicillin and gentamicin for seven days.

‡Arm B – oral amoxicillin and intramuscular gentamicin for seven days.

§Arm C – intramuscular procaine penicillin and gentamicin for two days followed by oral amoxicillin for another five days.

**¶**Including death till day 15, failure by day eight and non-fatal relapse between day nine and 15.

We randomised 2544 infants to study arm A, 2531 to arm B and 2542 to arm C in the three trials [9-11]. The baseline characteristics of all randomised infants in the three study arms (A, B, and C) were similar, reflecting the success of randomization (**Table 2**).

**Table 2:** Selected characteristics of participants by treatment arm*

Characteristics	Arm A†	Arm B‡	Arm C§
**Enrolled (N)**	2544	2531	2542
**Infants <7 days**	740 (29.1%)	724 (28.6%)	744 (29.3%)
**Male**	1468 (57.7%)	1398 (55.2%)	1376 (54.1%)
**Clinical signs at enrolment**			
One sign only	2009 (79.0%)	2009 (79.4%)	2011 (79.1%)
More than one	535 (21.0%)	522 (20.6%)	531 (20.9%)
High body temperature	1084 (42.6%)	1085 (42.9%)	1112 (43.7%)
Low body temperature	129 (5.1%)	133 (5.2%)	165 (6.5%)
Severe chest indrawing present	1170 (46.0%)	1156 (45.7%)	1139 (44.8%)
Stopped feeding well	664 (26.1%)	637 (25.2%)	629 (24.7%)
Movement only when stimulated	83 (3.3%)	90 (3.6%)	82 (3.2%)
**Weight 1500-2499 g**	384 (15.1%)	375 (14.8%)	393 (15.5%)
**Weight for age Z-score<-2**	724 (28.5%)	729 (28.8%)	734 (28.9%)
**Facility delivery**	1202 (47.2%)	1174 (46.4%)	1174 (46.2%)

*Data presented as n (%) unless otherwise specified.

†Arm A- intramuscular daily injections of procaine penicillin and gentamicin for seven days.

‡Arm B- oral amoxicillin and intramuscular gentamicin for seven days.

§Arm C- intramuscular procaine penicillin and gentamicin for two days followed by oral amoxicillin for another five days.

**Table 3** presents the risk difference and risk ratio for poor clinical outcomes, both for per-protocol and intention-to-treat analyses. Overall, for per-protocol analysis, 11.6% of infants in the reference regimen (arm A) had poor clinical outcomes by day 15, compared to 9.4% in arm B and 10.6% in arm C. The risk difference between arm A and arm B was -2.1% (95% CI = -3.8%, -0.4%; *P* = 0.016), and the risk ratio was 0.81 (95% CI = 0.69, 0.96). Similarly, for intention-to-treat analysis, 11.6% of 2544 infants in arm A, 9.8% of 2531 infants in Arb B, and 10.9% of 2542 infants in arm C had poor clinical outcomes. The risk difference between arm A and B was -1.8% (95% CI = -3.5%, -0.2%; *P* = 0.031), and the risk ratio was 0.84 (95% CI = 0.72%, 0.99%). Those in arm C did not have an increased risk of poor clinical outcome compared to arm A for both per-protocol (risk difference = -1.1%, 95% CI = -2.8%, 0.6%) and intention-to-treat (risk difference = -0.8%, 95% CI = -2.5%, 0.9%) analyses.

**Table 3:** Poor clinical outcome (death by day 15, treatment failure by day seven or non-fatal relapse between day eight and 15) by treatment arms for each trial and overall: A) per-protocol and B) intention to treat*

	Arm A†	Arm B‡	Arm C§	Risk difference (95% CI)		Risk ratio (95% CI)	
**A. Per-protocol**				**Arm A vs Arm B†‡**	**Arm A vs Arm C†§**	**Arm A vs Arm B†‡**	**Arm A vs Arm C†§**
**Overall**	276/2370 (11.6%)	223/2359 (9.4%)	254/2405 (10.6%)	-2.1% (-3.8%, -0.4%)¶**	-1.1% (-2.8%, 0.6%)¶††	0.81 (0.69, 0.96)¶	0.91 (0.77, 1.07)¶
AFRINEST	75/828 (9.1%)	58/826 (7.0%)	67/862 (7.8%)	-2.0% (-4.6%, 0.6%)	-1.3% (-3.9%, 1.4%)	0.77 (0.56, 1.08)	0.86 (0.62, 1.18)
Bangladesh trial	91/795 (11.4%)	79/782 (10.1%)	80/790 (10.1%)	-1.3% (-4.4%, 1.7%)	-1.3% (-4.4%, 1.7%)	0.88 (0.66, 1.17)	0.88 (0.67, 1.17)
Pakistan trial	110/747 (14.7%)	86/751 (11.4%)	107/753 (14.2%)	-3.3% (-6.7%, 0.1%)	-0.5% (-4.1%, 3.0%)	0.78 (0.60, 1.01)	0.96 (0.75, 1.23)
**B. Intention-to-treat**							
**Overall**	296/2544 (11.6%)	248/2531 (9.8%)	277/2542 (10.9%)	-1.8% (-3.5%, -0.2%)¶‡‡	-0.8% (-2.5%, 0.9%)¶§§	0.84 (0.72, 0.99)¶	0.94 (0.80, 1.09)¶
AFRINEST	80/894 (8.9%)	59/884 (6.7%)	69/896 (7.7%)	-2.3% (-4.8%, -0.2%)	-1.2% (-3.8%, 1.3%)	0.75 (0.54, 1.03)	0.86 (0.63, 1.17)
Bangladesh trial	96/830 (11.6%)	97/831 (11.7%)	87/829 (10.5%)	0.1% (-3.0%, 3.2%)	-1.1% (-4.1%, 1.9%)	1.0 (0.77, 1.32)	0.91 (0.69, 1.19)
Pakistan trial	120/820 (14.6%)	92/816 (11.3%)	121/817 (14.8%)	-3.3% (-6.6%, -0.1%)	0.2% (-3.2%, 3.6%)	0.77 (0.58, 0.99)	1.01 (080, 1.28)

CI – confidence interval, AFRINEST – African Neonatal Sepsis Trial

*Data presented as n/N (%) unless otherwise specified.

†Arm A: intramuscular daily injections of procaine penicillin and gentamicin for seven days.

‡Arm B: oral amoxicillin and intramuscular gentamicin for seven days.

§Arm C: intramuscular procaine penicillin and gentamicin for two days followed by oral amoxicillin for another five days.

¶Adjusted for trial

**P = 0.016.

††*P* = 0.214.

‡‡*P* = 0.031.

§§*P* = 0.337.

Overall, young infants who received arm B had a lower risk of poor clinical outcomes compared to the combined arms A and C for both per-protocol (risk difference = -1.6%, 95% CI = -3.0%, -0.1%; *P* = 0.035) and intention-to-treat (risk difference = -1.4%, 95% CI = -2.8%, -0.1%; *P* = 0.049) analyses (**Table 4**).

**Table 4:** Comparison of poor clinical outcome (death by day 15, treatment failure by day 7 or non-fatal relapse between day 8 and 15) between combined treatment arms A and C and arm B for each trial and overall: A) per-protocol and B) intention to treat

A. Per-protocol	Arm A* and C†	Arm B‡	Risk difference (95% CI)	Risk ratio (95%CI)
**Overall, – n/N (%) **	**530/4775 (11.1%)**	**223/2359 (9.4%)**	**-1.6%^§^(-3.0%, -0.1%)^¶^**	**0.85^§^(0.73, 0.98)**
AFRINEST – n/N (%)	142/1690 (8.4%)	58/826 (7.0%)	-1.4% (-3.6%, 0.8%),	0.84 (0.62, 1.12)
Bangladesh trial – n/N (%)	171/1585 (10.8%)	79/782 (10.1%)	-0.7% (-3.3%, 1.9%)	0.94 (0.73, 1.21)
Pakistan trial – n/N (%)	217/1500 (14.5%)	86/751 (11.5%)	-3.0% (-5.9%, -0.1%)	0.79 (0.63, 1.00)
**B. Intention-to-treat**				
**Overall, – n/N (%)**	**573/5086 (11.3%)**	**248/2531 (9.8%)**	**-1.4%† (-2.8%, -0.1%)^#^**	**0.87† (0.75, 0.99)**
AFRINEST – n/N (%)	149/1790 (8.3%)	59/884 (6.7%)	-1.6% (-3.7%, 0.4%)	0.80 (0.60, 1.07)
Bangladesh trial – n/N (%)	183/1659 (11.0%)	97/831 (11.7%)	0.6% (-2.0%, 3.3%)	1.06 (0.84, 1.33)
Pakistan trial – n/N (%)	241/1637 (14.7%)	92/816 (11.3%)	-3.4% (-6.2%, -0.6%)	0.77 (0.61, 0.96)

*Arm A – intramuscular daily injections of procaine penicillin and gentamicin for 7 days.

†Arm C – intramuscular procaine penicillin and gentamicin for 2 days followed by oral amoxicillin for another 5 days.

‡ Arm B – oral amoxicillin and intramuscular gentamicin for 7 days.

§Adjusted for the trial.

**¶**P-value: 0.035.

**#**P-value: 0.049.

## DISCUSSION

Both the per-protocol and intention-to-treat analyses showed that the risk difference and risk ratio were in the favour of the gentamicin-oral amoxicillin WHO-recommended regimen compared to the gentamicin-procaine penicillin regimen for outpatient treatment of young infants with PSBI when a referral is not feasible [13]. The individual AFRINEST [9] and SATT [10,11] trials reported equivalence between regimens, but pooling of the individual patient-level data provided the statistical power to detect superiority of one regimen over the other.

The definition of the outcome we used for the current analyses included death till day 15, treatment failure by day eight and relapse by day 15, which were all poor clinical outcomes in the individual trials. We believe clinical efficacy is the only feasible marker for comparing antibiotic regimens in settings where referral is not feasible. In AFRINEST and SATT studies [9-11], clinical signs were reliably measured during the 15-day follow-up after randomization by trained clinical assessors. The risk of measurement bias was reduced by using an outcome assessment team not involved in the provision of treatment to the infants in the AFRINEST [9]; the use of a second assessor to confirm findings in Bangladesh and Pakistan, and video documentation in a proportion of cases in Pakistan [15,17,18].

The simplified antibiotic treatment regimens in these studies are expected to be used in first-level facilities via the WHO Integrated Management of Common Illness (IMCI) tool [14], which utilises the clinical syndrome of PSBI. Laboratory investigations, particularly on blood cultures, are not available at these facilities. Even in high-resource settings, the empirical antibiotic treatment of suspected bacterial sepsis in sick neonates and young infants is primarily based on clinical signs [19,20]. However, in high-resource settings, antibiotic treatment is stopped after 48 hours if the culture is negative and the infant is well. In many low-resource settings where hospitalisation and laboratory tests are not feasible, antibiotic therapy is continued until completion [9-11,13].

The choice of these antibiotic regimens for outpatient use in the AFRINEST and SATT studies might be questioned by some. First, various antibiotic regimens had been used to manage neonatal sepsis at the community or an outpatient level in several research studies. Bang et al. [5] reported a 76% reduction in neonatal sepsis mortality in Gadchiroli, India, through community management of infection using injectable gentamicin plus oral cotrimoxazole. In Morang, Nepal, young infants classified as having PSBI were treated with oral cotrimoxazole and referred to a health facility for treatment with injectable gentamicin for seven days [21]. In Sylhet, Bangladesh, Baqui et al. [4] showed a 34% reduction in neonatal mortality using a community-based package of interventions, including intramuscular procaine penicillin and gentamicin, to treat suspected neonatal sepsis when referral to a hospital was not accepted. In Karachi, Pakistan, Zaidi et al. [6] compared three antibiotic regimens on an outpatient basis in young infants with PSBI whose families refused referral to a hospital. They compared injection ceftriaxone regimen, injection gentamicin plus oral trimethoprim-sulfamethoxazole regimen, and injection procaine penicillin plus gentamicin regimen. They reported that the penicillin-gentamicin regimen was the most effective (91% success rate), followed by ceftriaxone (85% success rate), whereas the trimethoprim-sulfamethoxazole and gentamicin regimen (82% success rate) had a significantly higher death rate compared to the other regimens. Second, the WHO, with other partners, convened a consultative meeting of neonatal health experts in 2007 to discuss community-based approaches for neonatal sepsis management when hospitalisation is not feasible in high neonatal mortality settings [22]. They recommended that various combination oral and injectable antibiotic regimens or injectable to oral “switch” regimens potentially feasible in weak health systems for use in first-level facilities should be evaluated. Additionally, they should be acceptable to families and show treatment success rates as close as possible to the current standard regimen of injection gentamicin and penicillin for 7-10 days [3]. Third, for empirical therapy of suspected sepsis, penicillin/ampicillin and an aminoglycoside (usually gentamicin) are used globally in the neonatal age group and the second month of life [3,19,20]. The combination of penicillin/amoxicillin and gentamicin targets common neonatal pathogens such as *Escherichia coli*, other enteric gram-negative rods, streptococci, and pneumococci. It was impractical to give injectable benzylpenicillin or ampicillin four times a day in an outpatient setting. Injection procaine penicillin plus gentamicin was selected as the reference arm to treat PSBI based on the recommendations from the WHO consultation, the pharmacologic profile of antibiotics [23,24], and usage experience [4-6]. Both can be given once daily and can successfully treat most bacterial infections in young infants. Fourth, oral amoxicillin was chosen for simplified antibiotic arms because of its safety, extensive use experience in neonates, and high bioavailability [25-28]. For community management of neonatal pneumonia, oral antibiotics had been used successfully to reduce pneumonia and neonatal mortality [29,30]. Fifth, third-generation cephalosporins such as ceftriaxone were not considered for these trials to reserve their use in the treatment of meningitis and as a second-line antibiotic in a hospital. In addition, Zaidi et al. [6] did not show it to be more effective than the penicillin-gentamicin combination, in vitro susceptibility data does not suggest third-generation cephalosporins are more effective in treating sepsis [31], and some concerns exist about ceftriaxone in neonates due to potential toxicity [32]. Finally, we should note that no study drug-related serious adverse events were reported in AFRINEST or SATT.

A potential explanation for procaine-penicillin being less effective than oral amoxicillin could be its slow dissolution after intramuscular injection, giving a maximum blood level at about 4 hours, which falls slowly over the next 15-20 hours [33]. A 50 000 unit/kg body weight intramuscular dose of procaine penicillin produces a lower 24-hour trough concentration of 0.4 μg/mL, due to their increased clearance and shorter half-lives in older neonates compared to the first week of life when the 24-hour trough concentration of 1.5 μg/mL [34]. There are also some practical problems with the use of the injection procaine penicillin. Zaidi et al. [6] reported difficulties in giving two injections (penicillin plus gentamicin) for seven days to small babies and that 10% of families withdrew from the study after the first day refusing all further injections. Extensive family counselling was needed to complete the seven-day course of two injections daily. Additionally, the procaine penicillin injections were difficult to mix, viscous, and hard to administer with narrow bore needles, as reported by their study physicians [6]. These difficulties have programmatic implications.

Soon after its launch, the 2015 WHO PSBI management guideline when referral is not feasible was implemented in several countries to learn lessons on how to use it in diverse contexts. Summary results showed that the estimated coverage of PSBI treatment (including inpatient and outpatient treatment) in sick young infants was 76% when the guideline was used [35]. If such a treatment option did not exist, then these sick young infants would have been deprived of treatment, and some would have died. The overall case fatality risk (CFR) from these implementation experiences was less than 2% for young infants with PSBI, which was much lower than the 9.8% reported in a systematic review [8,35].

Equivalence trials comparing antibiotic regimen options for treatment of sick young infants with PSBI when inpatient treatment is not feasible are criticized by some experts because they excluded clinically severe cases and did not confirm neonatal sepsis microbiologically [36-38]. If few participants had sepsis, the different antibiotic options would be likely to appear equivalent no matter what their real efficacy However, the study by Zaidi et al. [6], which had a similar design but some differences in eligibility criteria in Pakistan, compared three antibiotic regimens on an outpatient basis in young infants with PSBI whose families refused referral to a hospital. As mentioned earlier, they reported the procaine penicillin-gentamicin and Ceftriaxone regimens to be superior to trimethoprim-sulfamethoxazole. Moreover, in a double-blind, randomised trial, oral amoxicillin was found to be superior compared to a placebo in young infants with fast breathing only [39] This trial was stopped prematurely by the Data and Safety Monitoring Board because the superiority of amoxicillin was demonstrated over placebo, with two deaths occurring in the placebo group. This pooled analysis provides clear evidence that the simpler WHO-recommended regimen was superior to the comparison regimen, not just within equivalence margins suggested by individual trials, thus mitigating much of the criticism that equivalence was a foregone conclusion of the trials given the patient population.

The main strength of our analyses is the large sample size from both African and Asian countries, which helps its generalizability. Second, a pooled analysis at the individual patient level was conducted with this large multi-country data, which supports the robustness of our results. The main limitations of this analysis are those inherent to the original trials. First, these trials were not blinded because it was unethical to give placebo injections to sick young infants to have an equal number of injections in all arms. Second, microbiological or other laboratory tests were not used to support the clinical diagnosis, as it was not feasible to set up such services at the first-level health facilities within the available resources.

## CONCLUSIONS

Analysis of pooled individual patient-level data from three large trials in Africa and Asia showed that the WHO-recommended simplified antibiotic regimen B (oral amoxicillin and injection gentamicin) was superior to regimen A (injection procaine penicillin and injection gentamicin) and combined arms A and C (injection procaine penicillin and injection gentamicin, followed by oral amoxicillin) in terms of poor clinical outcome for the outpatient treatment of young infants with PSBI when inpatient treatment was not feasible. These findings can help to substantially expand our options to increase access to early and effective treatment for those who may otherwise go untreated.
